# The choice of a thermodynamic formulation dramatically affects modelled chemical zoning in minerals

**DOI:** 10.1038/s41598-021-97568-x

**Published:** 2021-09-21

**Authors:** L. Tajčmanová, Y. Podladchikov, E. Moulas, L. Khakimova

**Affiliations:** 1 Institute of Earth Sciences, Heidelberg University, Heidelberg, Germany; 2 Institute of Earth Science, University of Lausanne, Lausanne, Switzerland; 3 Faculty of Mechanics and Mathematics, Moscow State University, Moscow, Russian Federation; 4 Institute of Geosciences & Mainz Institute of Multiscale Modeling (M3ODEL), Johannes-Gutenberg University of Mainz, Mainz, Germany; 5 Skolkovo Institute of Science and Technology, Moscow, Russian Federation

**Keywords:** Solid Earth sciences, Mineralogy, Petrology, Structural materials, Theory and computation

## Abstract

Quantifying natural processes that shape our planet is a key to understanding the geological observations. Many phenomena in the Earth are not in thermodynamic equilibrium. Cooling of the Earth, mantle convection, mountain building are examples of dynamic processes that evolve in time and space and are driven by gradients. During those irreversible processes, entropy is produced. In petrology, several thermodynamic approaches have been suggested to quantify systems under chemical and mechanical gradients. Yet, their thermodynamic admissibility has not been investigated in detail. Here, we focus on a fundamental, though not yet unequivocally answered, question: which thermodynamic formulation for petrological systems under gradients is appropriate—mass or molar? We provide a comparison of both thermodynamic formulations for chemical diffusion flux, applying the positive entropy production principle as a necessary admissibility condition. Furthermore, we show that the inappropriate solution has dramatic consequences for understanding the key processes in petrology, such as chemical diffusion in the presence of pressure gradients.

## Introduction

Chemical reactions and phase transformations under non-hydrostatic conditions play an important role in many industrial applications as well as natural processes^1,2^. Despite their abundance, there are still controversies related to the appropriate thermodynamic formulation, which attract significant research interests from experimental as well as theoretical points of view^3,4^.

In rock systems, we commonly deal with an apparent disequilibrium at all scales (i.e. from kilometer-meter at outcrop scale to micrometer-nanometer scale in thin sections). On the grain scale (mm-µm), such observations are represented by a chemical zoning or mineral assemblages that are not in equilibrium. They are traditionally interpreted as a result of sluggish chemical diffusion, i.e. so-called diffusion-controlled process. Additionally, mechanically-imposed gradients in pressure or various stress components develop in materials due to deformation. These, so called, mechanically-controlled systems can be documented by direct measurements of variations in residual strain, and in turn stress and pressure^5,6^. Moreover, the measured residual pressure variations have been progressively reported using the inclusion elastic barometry method^7–9^. This direct evidence has augmented the need for pressure/stress variations to be considered in the petrological quantifications^10,11^. In fact, the quantifications of rock microstructures involving both, the chemical and mechanical, gradients are the gateway to the accurate description of complex processes in geological systems and the development of predictive models. Interestingly, the quantification of such a complex system is still ambiguous.

The magnitude of pressure/stress variations in rocks and its effect on chemical processes has been a major focus of research over the last few years^12–14^. Several works pointed out that the chemical zoning in minerals can be mechanically-controlled either as equilibrium under pressure gradients^13^, or as a coupled, chemical diffusion and mechanical deformation process^15^. In these works, all thermodynamic formulations, that describe the system under pressure gradients, involve mass units following the classical physics and transport phenomena literature^16–19^. Interestingly, this triggered a fundamental discussion on the necessity of using mass-units approach highlighting the convenience of molar approach in petrological calculations^20^. In practice, both mass-based and molar-based formulations are used, with the first being preferred in silicate melts^21^ and the latter commonly used in silicate minerals^22^.

Here, we show that, apart from convenience, there is another reason to be considered before choosing the preferred formulation. In the absence of a specific experiment which would prove the correct thermodynamic solution, the entropy production principle is the only way how to evaluate the thermodynamic admissibility. A thermodynamically admissible solution has a non-negative contribution of all the dissipative thermo-mechanical-chemical processes to the entropy production^23^. Here, we evaluate the admissibility of the thermodynamic formulations for chemical diffusion flux in mass and molar units with respect to the entropy production during the transient process on the way to the equilibrium state. First, we compare both formulations mathematically to see whether they guarantee the positive entropy production criterion. Furthermore, we apply both formulations to mechanically-controlled plagioclase binary system, where we quantify the entropy production and explore the petrological consequences of potentially invalid thermodynamic procedure.

### Entropy production—the decision maker

The admissibility of the mass or molar thermodynamic formulation commonly used for rock microstructures cannot be unequivocally justified for a system at local equilibrium where the chemical diffusion flux is equal to zero. Therefore, we focus on the process prior to the equilibrium stage and investigate how the equilibrium state is established. We consider the chemical diffusion flux by allowing pressure gradients in solids. Thermodynamics under pressure gradients is well established for fluids, including equilibrium for fluid mixtures under gravity field, as well as non-equilibrium thermodynamics for shock waves^24^. Importantly, a similar thermodynamic approach is successful to quantify shock waves in solids^25,26^ despite the inevitably large deviatoric stresses developing during the shock-wave deformation process. Specifically, we use Classical Irreversible Thermodynamics (labelled as CIT in Lebon et al.^27^) that is applicable to gases, fluids and solids. Solids with negligible influence of deviatoric stresses are thermodynamically equivalent to fluids. Concerns were raised about whether this approach is suitable for solids with large deviatoric stresses^20,28^.

In fact, three other non-equilibrium thermodynamics theories have been developed in last 60 years to improve the apparent simplifying assumptions made in the CIT. One extension of CIT is dedicated to corrections applicable to the light-speed limit or relativistic effects, so called “Extended Irreversible Thermodynamics” (e.g. Lebon et al.^27^). In our application, the correction for the relativistic effects is negligible, therefore this extension is not considered. The second attempt to improve CIT, so called “Rational Thermodynamics”, questions the validity of the local thermodynamic equilibrium assumption of CIT for non-equilibrium processes^27,29^, that is imposed by the requirement of validity of Gibbs equation. Instead, Rational Thermodynamics attempts to derive the Gibbs equation using other principles, i.e. the Coleman-Noll procedure for heat-conducting viscous materials^30^. Unfortunately, the popularity of Rational Thermodynamics faded away after it produced thermodynamically unstable models for visco-elastic media (e.g. p. 182 in Mueller and Weiss^31^). Nevertheless, the Coleman-Noll procedure is further used in the modern thermodynamics treatments for solids (e.g. Equation 70.8 in Gurtin et al.^32^ is Gibbs equation derived via Colemann-Noll procedure). The third improvement of CIT is called “GENERIC” formalism^33,34^.

In relation to the multicomponent diffusion problem considered here, Truesdell^35^, the proponent of Rational Thermodynamics, established the equivalence of all the available approaches. Furthermore, in the most recent book on transport phenomena of Venerus & Öttinger^19^, the proponents of GENERIC formalism, multicomponent diffusion is treated in a fully CIT way. Moreover, both the CIT and the modern thermodynamics treatments^32^ provide mathematically identical expressions for entropy production, as shown below (Eq. 1). Importantly, if any effect is excluded from the model formulation by being not relevant for a given application, such as dependence of chemical potential on deviatoric elastic strain^32,36^, it does not violate any laws of nature, such as entropy production.

Provided the facts listed above, the CIT approach was chosen as the most appropriate formulation for the aim of this study. The model captures all essential physics and excludes negligible contributions from physically existing effects, such as external forces (e.g. gravity) or deviatoric elastic strain. Deviatoric elastic strain is not considered as a significant contribution to internal energy because it has been shown that the elastic strain correction for minerals is smaller than the accuracy of the currently available thermodynamic data^37^. Nevertheless, we considered this effect in Supplementary materials S1 and S2 for completeness.

In this part, we explore the mathematical validity of a mass vs. molar formulation. We focus only on the chemical potential part as a driving force for diffusion and ignore other sources of entropy production (e.g. thermal and mechanical). The complete derivation is provided in Supplementary material S1. Following the classical literature^16^, the entropy production ${Q}^{s}$ corresponds to

1 $${Q}^{s}=-\frac{{q}_{i}^{{\mathrm{x}}^{a}}}{T}\frac{\partial }{\partial {x}_{i}}\left(\frac{{\widehat{\mu }}^{a}}{{M}^{a}}-\frac{{\widehat{\mu }}^{\beta }}{{M}^{\beta }}\right)\ge 0$$

where ${q}_{i}^{{\mathrm{x}}^{a}}$ is the corresponding flux of component $a$ in the *i*th direction (in kg/(m^2^ s)), $\widehat{\mu }$ is the corresponding chemical potential (in J/mol) and $M$ is the molecular mass of the respective species (in kg/mol). The use of “hat” symbol (^) indicates mol-specific properties. In agreement with the second law of thermodynamics, the entropy production must be nonnegative (e.g. $\sigma$ in the notation of de Groot and Mazur^16^, p. 22). A mathematically equivalent expression to Eq. 1 using CIT can also be obtained by the Coleman-Noll procedure (Eq. 70.7 in Gurtin et al.^32^). This means that both, the classical irreversible and the most modern thermodynamics approaches, result in identical expression to ensure that the second law of thermodynamics is not violated.

For a chemical system under the presence of gradients the chemical flux is commonly defined as^19^:

2 $${q}_{i}^{{\mathrm{x}}^{a}}=-K\frac{\partial }{\partial {x}_{i}}\left(\frac{{\widehat{\mu }}^{a}}{{M}^{a}}-\frac{{\widehat{\mu }}^{\beta }}{{M}^{\beta }}\right)$$

where $K$ is a phenomenological coefficient that is determined by experiments. *K* is not a classical diffusivity coefficient because it is related to chemical potential and not concentration gradients. Therefore, the units of *K* are such to guarantee the dimensional consistency of the flux equation (in kg^2^/(m J s)). Following the classical approach in non-equilibrium thermodynamics, the only requirement for $K$ is that it has to be positive to satisfy the entropy production (${Q}^{s}$) condition (see Supplementary material S1). The positive $K$ drives the system to equilibrium and guarantees that diffusion eventually homogenizes diffusional chemical potential gradients. Substituting the flux ${q}_{i}^{{\mathrm{x}}^{a}}$ from Eq. 2 into Eq. 1, the entropy production expression becomes

3 $${Q}^{s}=\frac{K}{T}{\left[\frac{\partial }{\partial {x}_{i}}\left(\frac{{\widehat{\mu }}^{a}}{{M}^{a}}-\frac{{\widehat{\mu }}^{\beta }}{{M}^{\beta }}\right)\right]}^{2}$$

From the mathematical point of view, it is evident that this expression is non-negative due to the square of the gradient in diffusional chemical potential, in this case defined in mass units.

If we follow an arbitrary definition of the chemical flux, now using molar-based chemical potentials, we get

4 $$ q_{i}^{{{\text{x}}^{a} }} = - K^{\prime } \frac{\partial }{{\partial x_{i} }}\left( {\hat{\mu }^{a} - \hat{\mu }^{\beta } } \right) $$

where again $K^{\prime}$ is a positive constant to satisfy the requirements for Eq. (1) above. Using Eq. (4), the entropy production Eq. 1 becomes:

5 $$ Q^{s} = \frac{{K^{\prime } }}{T}\frac{\partial }{{\partial x_{i} }}\left( {\frac{{\hat{\mu }^{a} }}{{M^{a} }} - \frac{{\hat{\mu }^{\beta } }}{{M^{\beta } }}} \right)\frac{\partial }{{\partial x_{i} }}\left( {\hat{\mu }^{a} - \hat{\mu }^{\beta } } \right) $$

Despite the positive $K^{\prime }$ coefficient, the nonnegativity of the entropy production is not guaranteed. This is because the chemical potential differences are defined in an inconsistent form, i.e. the first in mass (as defined in Eq. 1) and the second in moles (from Eq. 4).

It is evident that the thermodynamic formulation of the chemical flux is always admissible in mass units. However, in the molar expression, the positive entropy production is not mathematically assured because the expression Eq. (5) can have both, positive or negative, results. Despite the fact that both Eqs. (2) and (4) for chemical potential would predict zero net flux at equilibrium, Eq. (4) might produce thermodynamically inadmissible results during the transient processes on the way to equilibrium. In fact, it questions whether the equilibrium state would be even reached using the molar formulation for the inadmissible cases, because such a path would be forbidden by the fundamental thermodynamic principles. Therefore, this mathematical validation can serve as tool to prove the admissibility of any rock-microstructure quantification, i.e. the positive entropy production principle.

### Example

**Figure 1:**
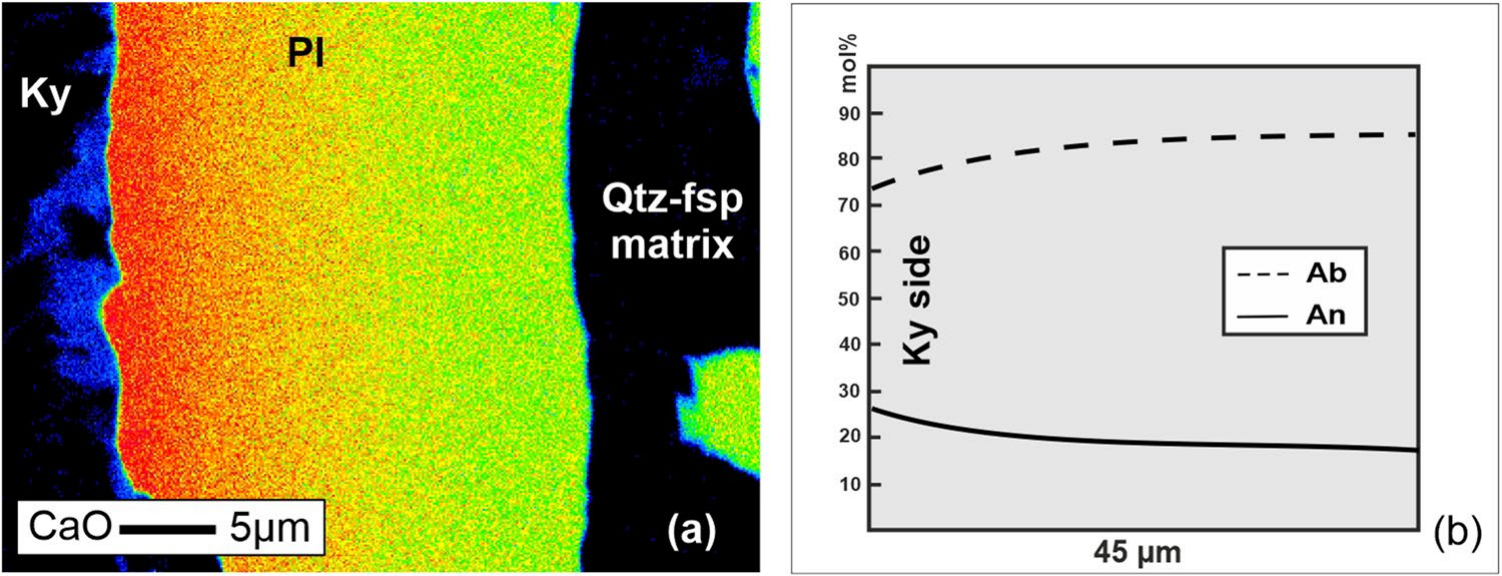
An example of a chemically-zoned plagioclase grain. (**a**) The element distribution map where the color-coding corresponds to the variation in CaO. (**b**) Representative compositional profile (line scan) across the plagioclase grain. Pl = plagioclase, Ky = kyanite, Qtz = quartz, fsp = feldspar, Ab = albite component in mol% ((Na/(Ca + Na)) × 100), An = anorthite component in mol % ((Ca/(Ca + Na)) × 100).

Using the mathematical argument derived in the previous section, we explore a simple binary natural case to identify the potentially inappropriate thermodynamic solution for a given system. We focus on an illustrative example of plagioclase, the most common mineral phase in the Earth’s crust. The microstructure with chemically zoned plagioclase rim around kyanite relic (Fig. 1) from felsic granulite of the Bohemian Massif in Czech Republic^38,39^ was selected as a representative sample for our test. The chemical zoning (Fig. 1) was interpreted to reflect the pressure gradients across the mechanically-controlled microstructure^13,40^. Interestingly, this thermodynamic formulation, namely the use of mass units, was questioned by Powell et al.^20^. Therefore, this microstructure provides an exceptional example to validate both, molar and mass, approaches for the given system.

#### Model setting

To investigate the evolution of the concentration profile and entropy production for a mechanically-controlled microstructure on the way to equilibrium (i.e. towards its steady state), a 1-D explicit finite difference numerical model was developed. No-flux (Neumann) boundary conditions from both sides of the mineral grain were assumed. The modelling was performed under constant temperature of 800 °C. The pressure difference across the plagioclase grain used in this illustrative model is 0.8 GPa, which is a value that was suggested by Tajčmanová et al.^13^. The thermodynamic database of Holland and Powell^41^ was used for the calculation of chemical potentials and plagioclase was considered as both, ideal and non-ideal solution^42^ for comparison.

The flux equation has two main contributors, the diffusion constant and the chemical potential difference. The magnitude of the positive constants ($K$ or $K^{\prime}$) in the diffusion flux equations would affect only the magnitude of the entropy production but not its sign. Since we do not use the model for diffusion geospeedometry/geochronometry purposes, the dependency of this constant can be eliminated by considering steady state, where the flux becomes zero. Provided that $K$ is positive, the flux direction, and thus the resulting concentration gradient, is only controlled by the chemical potential difference ($\frac{{\hat{\mu }^{a} }}{{M^{a} }} - \frac{{\hat{\mu }^{\beta } }}{{M^{\beta } }}$ or $\hat{\mu }^{a} - \hat{\mu }^{\beta }$) and its sign. Therefore, the value of the constant ($K$ or $K^{\prime }$) is arbitrary in the model, as described in relation to Eq. (2).

The following general expression for chemical potential is considered

6 $$ \hat{\mu }^{a} = \hat{\mu }_{P,T}^{a} + RTln\left( { a^{\alpha } } \right) $$

where $R$ is the universal gas constant, $T$ is temperature, ${a}^{\alpha }$ is the activity of $\alpha$ in the solid solution and ${{\widehat{\mu }}_{P,T}}^{a}$ is equal to the molar Gibbs energy of the mineral endmember that needs to be integrated from reference conditions. In the absence of temperature gradient, the gradient of chemical potential can be written as

7 $$ \frac{{\partial \hat{\mu }^{a} }}{{\partial x_{i} }} = \frac{{\partial \hat{\mu }^{a} }}{\partial P}\frac{\partial P}{{\partial x_{i} }} + RT\frac{{\partial ln\left( { a^{\alpha } } \right)}}{{\partial x_{i} }} = {\hat{\text{v}}}^{a} \frac{\partial P}{{\partial x_{i} }} + \frac{RT}{{a^{\alpha } }}\frac{{\partial a^{\alpha } }}{{\partial x_{i} }} $$

where ${\widehat{\mathrm{v}}}^{a}$ is the molar volume of the end-member phase $a$. Using a molar formulation, the gradient of chemical potential difference becomes

8 $$ \frac{{\partial \left( {\hat{\mu }^{a} - \hat{\mu }^{\beta } } \right)}}{{\partial x_{i} }} = \left( {{\hat{\text{v}}}^{a} - {\hat{\text{v}}}^{\beta } } \right)\frac{\partial P}{{\partial x_{i} }} + RT\left( {\frac{1}{{a^{\alpha } }}\frac{{\partial a^{\alpha } }}{{\partial x_{i} }} - \frac{1}{{a^{\beta } }}\frac{{\partial a^{\beta } }}{{\partial x_{i} }}} \right) $$

The equivalent expression using mass-specific properties is:

9 $$ \frac{{\partial \left( {\frac{{\hat{\mu }^{a} }}{{M^{a} }} - \frac{{\hat{\mu }^{\beta } }}{{M^{\beta } }}} \right)}}{{\partial x_{i} }} = \left( {\frac{{{\hat{\text{v}}}^{a} }}{{M^{a} }} - \frac{{{\hat{\text{v}}}^{\beta } }}{{M^{\beta } }}} \right)\frac{\partial P}{{\partial x_{i} }} + RT\left( {\frac{1}{{M^{a} a^{\alpha } }}\frac{{\partial a^{\alpha } }}{{\partial x_{i} }} - \frac{1}{{M^{\beta } a^{\beta } }}\frac{{\partial a^{\beta } }}{{\partial x_{i} }}} \right) $$

By using density of the end members ($\rho^{a,\beta }$), Eq. (9) can be rewritten as follows:

10 $$ \frac{{\partial \left( {\frac{{\hat{\mu }^{a} }}{{M^{a} }} - \frac{{\hat{\mu }^{\beta } }}{{M^{\beta } }}} \right)}}{{\partial x_{i} }} = \left( {\frac{1}{{\rho^{a} }} - \frac{1}{{\rho^{\beta } }}} \right)\frac{\partial P}{{\partial x_{i} }} + RT\left( {\frac{1}{{M^{a} a^{\alpha } }}\frac{{\partial a^{\alpha } }}{{\partial x_{i} }} - \frac{1}{{M^{\beta } a^{\beta } }}\frac{{\partial a^{\beta } }}{{\partial x_{i} }}} \right) $$

Results for both, ideal and non-ideal solutions for plagioclase, were compared to capture the discrepancies related to the solid solution model configuration. The compositional part in the chemical potential expression (Eq. 6) was positive, regardless whether the mass or molar formulation is used. The dramatic difference between the Eqs. (8) and (10) is the factor multiplied with the pressure gradient. This factor plays an essential role in the quantification of the systems under pressure gradients as suggested in Ref.^13,40^. In the model results below, we show, how the chemical potential difference can be significantly different under the presence of pressure gradients, depending on the sign and magnitude of this factor.

#### Results

**Figure 2:**
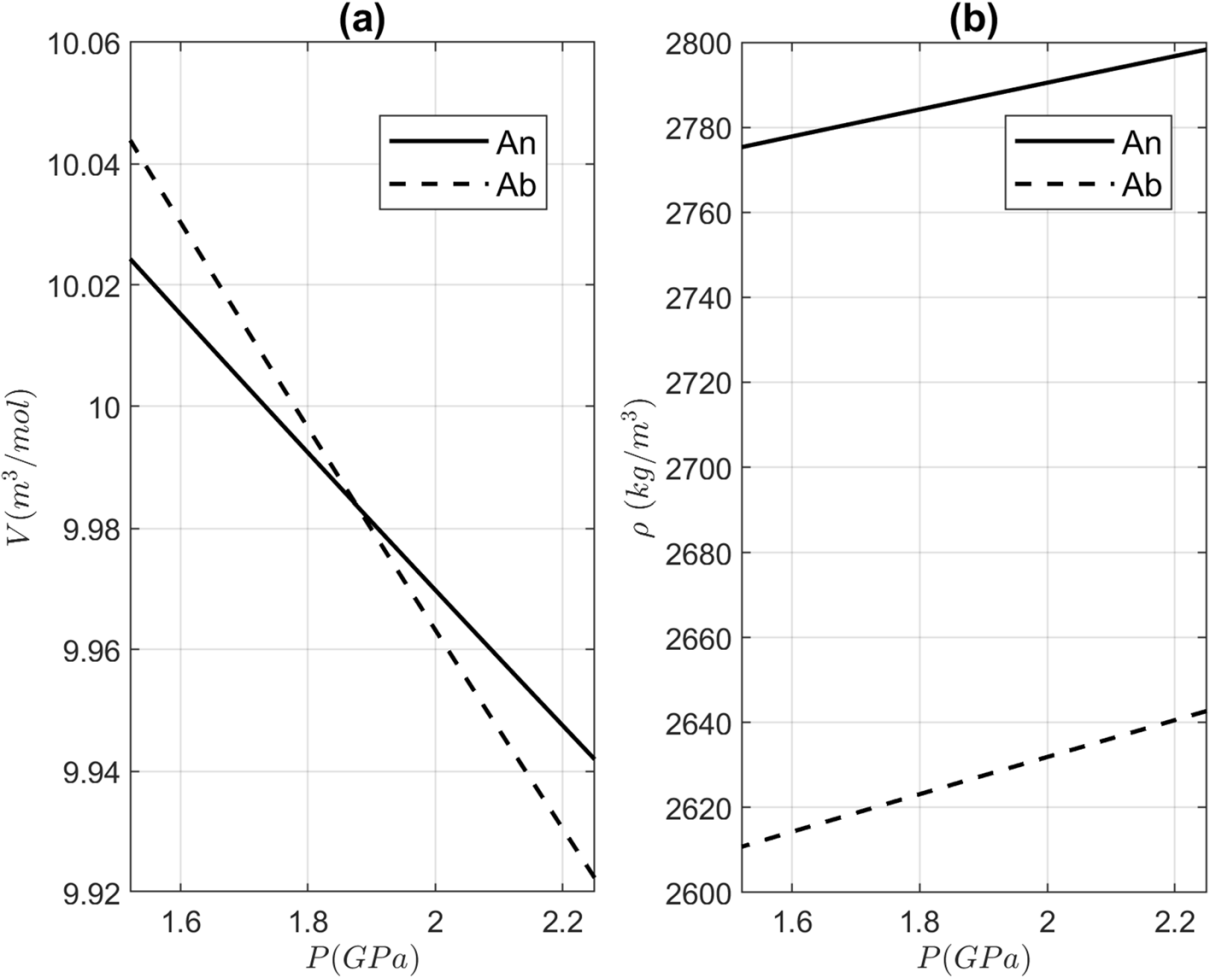
(**a**) Molar volumes (J/mol/bar) and (**b**) density (kg/m^3^) of albite (Ab) and anorthite (An) endmembers as a function of pressure (*P*).

Molar volumes and densities of the plagioclase endmembers, albite and anorthite, have significantly different trends (Fig. 2). We use the cross-over in the molar volume of both endmembers at around 1.88 GPa (Fig. 2a) as an apparent threshold in our analysis.

**Figure 3:**
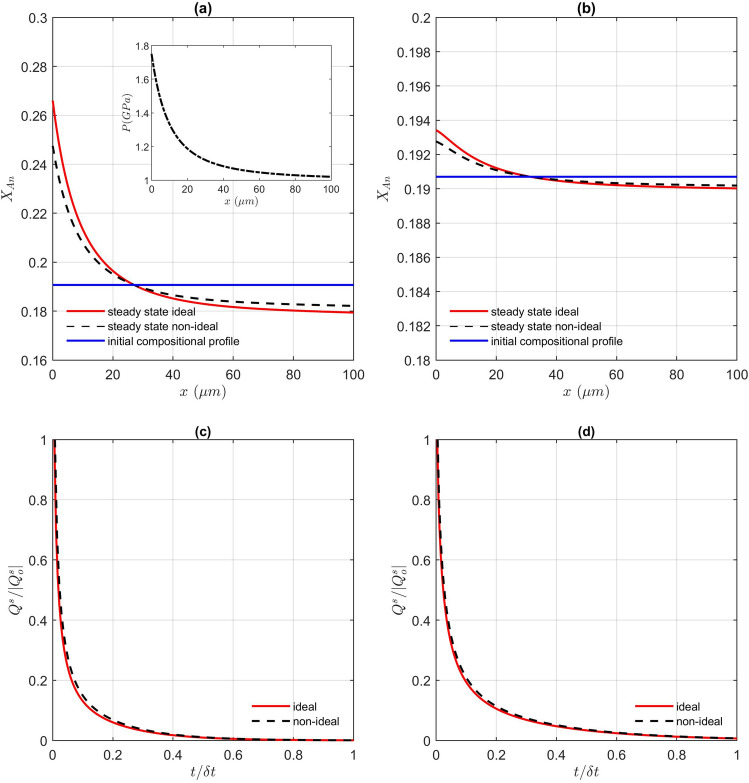
Resulting compositional profile (X Anorthite = Ca/(Ca + Na)) as a funcion of distance for (**a**) mass and (**b**) molar solution. The pressure profile assumed (1.8 to 1 GPa) is shown as inset in a). Evolution of entropy production with time (both normalized) for the same pressure variation using the diffusion flux in (**c**) Mass (Eq. 2) and (**d**) Moles (Eq. 4). Both, ideal and non-ideal^42^ plagioclase solutions are compared.

We first investigated the evolution of the compositional profile across the plagioclase grain for the given pressure profile below the cross-over in molar volumes at 1.88 GPa (Fig. 2a), using Eqs. 2 (mass) and 4 (moles) (Fig. 3). Due to the functional dependence of the chemical potential on pressure described above, the diffusion flux is set in the initially chemically homogeneous plagioclase grain with mechanically-imposed pressure gradient. Under this pressure gradient, the diffusion process leads to the development of a chemical zoning on the way to equilibrium. The final compositional profile at given time in this model corresponds to the steady state, where the mass fluxes are balanced and concentrations remain unchanged. Under these conditions, the diffusion flux is zero but the plagioclase grain is chemically zoned due to the mechanically-imposed pressure gradient. When both profiles, mass (Fig. 3a) and molar (Fig. 3b), are compared, the trend of the steady state chemical zoning is similar for both, whereas the magnitude is about one order of magnitude different. The trend of the entropy production is for both, mass and molar solution, positive, i.e. both solutions are apparently admissible (Fig. 3c,d). The change from an ideal and a non-ideal solution does not affect the final result (Fig. 3; Supplementary material S2). However, such a difference between the mass and the molar approach in prediction of the chemical zoning would be critical for a correct application of petrological approaches and interpretations. Due to such a significant difference in the predicted magnitude of the chemical trend, only one of these two solutions can correctly predict the natural observations. The question now is, which of the two is correct?

**Figure 4:**
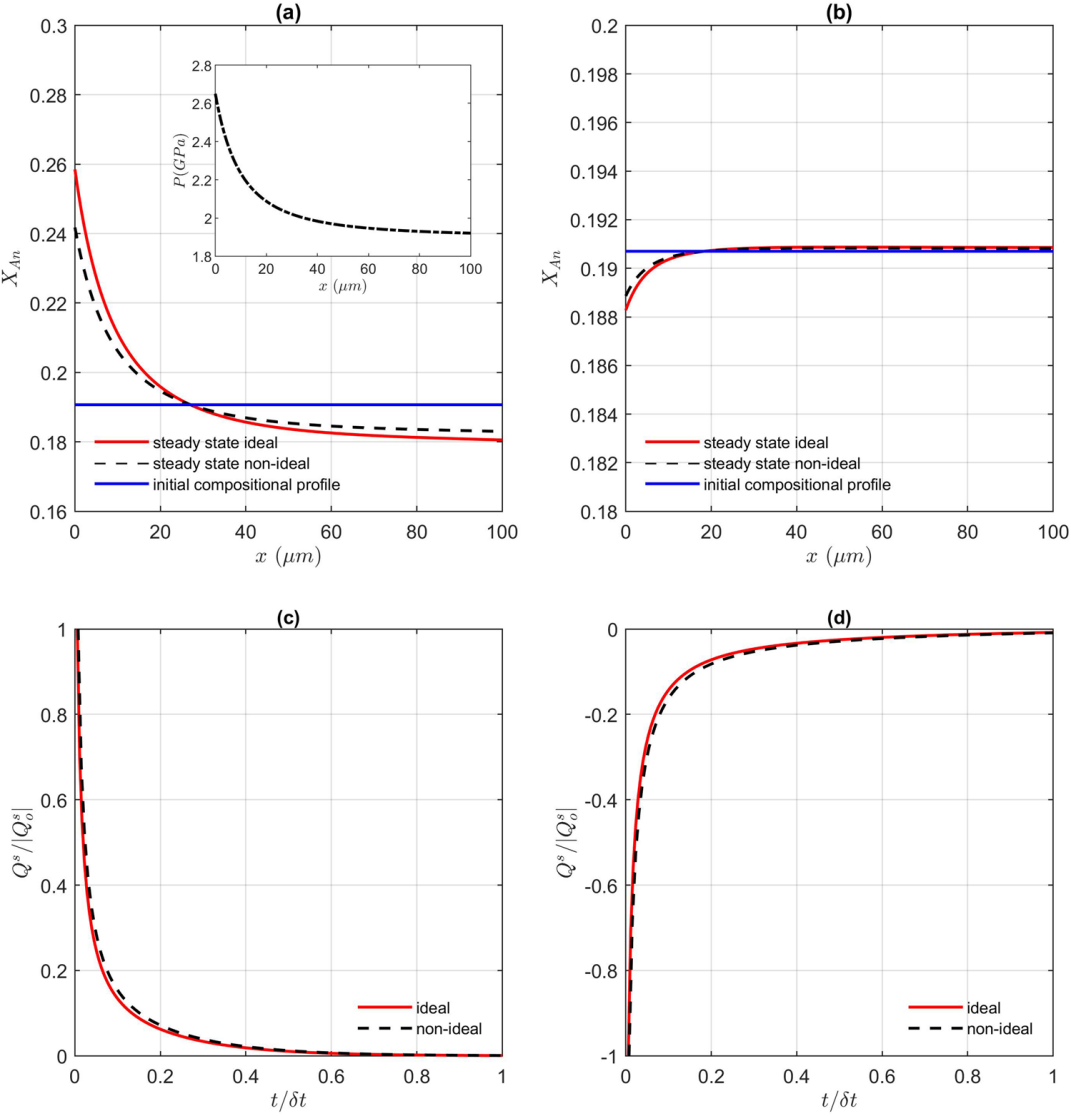
Resulting compositional profile (X Anorthite = Ca/(Ca + Na)) as a function of distance for (**a**) mass and (**b**) molar solution. The pressure profile assumed (2.7 to 1.9 GPa) is shown as inset in a). Evolution of entropy production with time (both normalized) for the same pressure variation using the diffusion flux in (**c**) Mass (Eq. 2) and (**d**) Moles (Eq. 4). Both, ideal and non-ideal^42^ plagioclase solutions are compared.

Interestingly, the molar trend of the chemical zoning above the 1.88 GPa threshold is completely opposite than the chemical trend inferred from the mass formulation (Fig. 4a,b). When we look at the entropy production contribution from chemical diffusion, it is positive for the flux formulation in mass (Eq. 2; Fig. 4c). This was expected from the mathematical point of view, due to the square of the gradient in chemical potential in Eq. 3. On the contrary, the entropy production is negative for the molar solution (Eqs. 4, 5; Fig. 4d). As in the previous test, the change from an ideal and a non-ideal solution does not have a significant effect on the final result (Fig. 4; Supplementary material S2). In fact, the trend of the entropy production in the molar solution (Fig. 4d) even questions whether equilibrium stage would be achieved during such a process. This is a documentation that the molar formulation for systems under pressure gradients is thermodynamically inconsistent and any attempt of supporting such results requires a rigorous demonstration of their thermodynamic admissibility.

## Discussion and conclusions

The direct documentation of stress/pressure variations in rock samples via elastic barometry, high-angular resolution electron backscatter diffraction (HR-EBSD) and X-ray microdiffraction has become very popular in geosciences. This augmented the need to understand the complex chemo-mechanical processes in rock systems and thus be able to quantify them.

We documented that for any thermodynamic formulation that is suggested to quantify a geological problem, it is essential to evaluate its admissibility following the entropy production principle. This is especially critical for systems under gradients. No matter how intuitive or comfortable the quantification is, if it contradicts the fundamental principles, such as positive entropy production, it is flawed. Furthermore, our analysis shows that the ideality vs. non-ideality of the solution model plays only a negligible role compared to the dramatic effect related to the choice of units (see also Supplementary material S2). The mass approach always guarantees that a solid solution under pressure gradient develops a chemical zoning such that the density of the mixture is distributed following the pressure gradient consistently. This result is not surprising because it is in agreement with the classical works on transport phenomena and non-equilibrium thermodynamics^16,18,24,43^. In fact, the classical works on transport phenomena have been extensively and thoroughly tested in numerous scientific and industrial applications, thus, the ad-hoc unit conversion without the theoretical demonstration of thermodynamic admissibility can have serious consequences.

Interestingly, following the molar approach, such as that proposed by Powell et al.^20^, this purely density-based distribution of the components in the system is not always guaranteed even for a classical example, where fluids under gravity are considered, such as stratified atmosphere or a glass of water. The inconsistency is related to the assumption that at equilibrium, the molar expression of chemical potential can be obtained by the multiplication of the mass-based chemical potentials with a constant molecular mass referring to Gibbs^44^ (Eq. 388, p.194). However, Gibbs^44^ in this part of his work considered a unary system, which is not applicable to diffusion in multicomponent systems as explained in Supplementary material S1 (see “Additional comment” in S1).

A thermodynamically admissible molar equivalent for a system under gradients is not excluded^17,23^. However, any proposed molar formulation must be appropriately re-derived from the first principles (i.e. following the logic of the Supplementary material S1) and validated with respect to entropy production. In fact, if a new model system is explored, it is always advisable to perform the calculations in mass description. Once the behavior of the system is understood for the mass formulation, a valid molar solution can be derived.

Complementary to the non-equilibrium thermodynamics approach used in this work, there are other thermodynamic approaches for solids, pioneered by Larché and Cahn^36^. Here, the aim was to compare the molar and mass non-equilibrium formulations and identify the admissible solution for systems under gradients in general. The discussion, whether Larché and Cahn theory is more appropriate than the classical non-equilibrium thermodynamics approach, is beyond the scope of this work. In fact, the currently available internally consistent thermodynamic datasets for minerals^41^ and activity models do not include corrections for deviatoric elastic strain. In order to make those datasets consistent with the Larché and Cahn theory, the energetic contributions from the strain tensor would have to be introduced for each end member. Therefore, there is no possibility to evaluate this effect in a fully quantitative way at the moment. Furthermore, minerals are commonly anisotropic, which adds even higher complexity to the problem, because the current datasets treat all minerals as isotropic. Nevertheless, we provide a comparison of the results presented here with respect to Larché and Cahn theory in Supplementary material S2. The generalization of chemical potential in the framework of thermodynamics of stressed solids by Larché and Cahn^36^ affects the magnitude of the compositional differences. However, the trend of chemical zoning remains the same as in our reference model, i.e. there is a significant difference between the mass and molar formulation. Therefore, this finding even highlights the importance of the appropriate choice of flux formulation.

The example showed here is for the plagioclase solid solution, the most abundant phase in the Earth’s crust. The cross-over in the molar volume of albite and anorthite endmembers of plagioclase at ca. 1.88 GPa leads to radical changes of the chemical trend in comparison to the mass approach (Fig. 2). Interestingly, such a difference between molar volume and mass can also be found for the grossular and pyrope endmembers in garnet^40^, the key mineral phase in metamorphic petrology. Therefore, an inappropriate solution can have dramatic consequences for understanding of the key processes in petrology, such as chemical diffusion in the presence of pressure gradients.

In general, the cross-over is clearly a critical factor that discriminates between mass and molar formulation. The most notable difference between Eqs. 8 and 10 is the factor multiplied with the pressure gradient. Under the pressure gradient, the chemical potential difference can take different values depending on the sign and magnitude of this factor. In principle, this would not be a problem, if it was just a unit conversion issue. However, as shown for the case of plagioclase, the difference of molar volumes $\left({\widehat{\mathrm{v}}}^{\mathrm{a}}-{\widehat{\mathrm{v}}}^{\upbeta }\right)$ and the difference of the density inverse $\left(\frac{1}{{\uprho }^{\mathrm{a}}}-\frac{1}{{\uprho }^{\upbeta }}\right)$ can have a different sign depending on the pressure, where they are evaluated. This means that the two choices of the diffusion flux result in opposite predictions with respect to the diffusion direction and, consequently, the final (steady state) trend of the chemical zoning.

Since the molar formulation does not guarantee the admissible solution, there might be other situations, where the molar formulation would give negative entropy production. Clearly, the final result is the interplay between the pressure and the compositional part in the chemical potential expression (Eq. 6). The cross-over in the molar volumes of individual endmembers seems to be the critical factor for the present case with large density differences between endmembers. Pressure gradients in systems with negligible density differences may not have such a large effect. Furthermore, based on the non-ideal solid solution behavior investigated in this work, the effects of non-ideality were not important. However, different formulations with a larger degree of non-ideality may lead to a different sign in the molar formulation at the specific conditions. These effects need to be explored in future applications.

## Supplementary Information

Supplementary Information 1.

Supplementary Information 2.

## Data Availability

Details on modelling and the approach are included in the main text. Additional material, as referred in the main text, is available in the Supplementary material. Further correspondence and requests for materials and codes should be addressed to L.T. (lucataj@gmail.com).

## References

[1] Feng B, Levitas VI (2017). Plastic flows and strain-induced alpha to omega phase transformation in zirconium during compression in a diamond anvil cell: finite element simulations. Mater. Sci. Eng. A.

[2] Levitas VI (2018). High pressure phase transformations revisited. J. Phys. Condens. Matter.

[3] Zhang L, Li Y-H, Gu Y-Q, Cai L-C (2019). Understanding controversies in the α-ω and ω-β phase transformation of zirconium from nonhydrostatic thermodynamics. Sci. Rep..

[4] Guńka PA, Olejniczak A, Fanetti S, Bini R, Collings IE, Svitlyk V, Dziubek K (2021). Crystal structure and non-hydrostatic stress-induced phase transition of urotropine under high pressure. Chem. Eur. J..

[5] Chen K, Kunz M, Tamura N, Wenk HR (2015). Residual stress preserved in quartz from the San Andreas Fault Observatory at Depth. Geology.

[6] Wallis D (2020). Dislocation Interactions during low-temperature plasticity of olivine and their impact on the evolution of lithospheric strength. Earth Planet. Sci. Lett..

[7] Alvaro M, Mazzucchelli ML, Angel RJ, Murri M, Campomenosi N, Scambelluri M, Nestola F, Korsakov A, Tomilenko AA, Marone F, Morana M (2020). Fossil subduction recorded by quartz from the coesite stability field. Geology.

[8] Zhong X, Moulas E, Tajčmanová L (2020). Post-entrapment modification of residual inclusion pressure and its implications for Raman elastic thermobarometry. Solid Earth.

[9] Moulas E (2020). Calculating pressure with elastic geobarometry: A comparison of different elastic solutions with application to a calc-silicate gneiss from the Rhodope Metamorphic Province. Lithos.

[10] Moore J, Beinlich A, Austerheim H, Putnis A (2019). Stress orientation–dependent reactions during metamorphism. Geology.

[11] Putnis A, Moore J, Prent AM, Beinlich A, Austrheim H (2021). Preservation of granulite in a partially eclogitized terrane: metastable phenomena or local pressure variations?. Lithos.

[12] Wheeler J (2014). Dramatic effects of stress on metamorphic reactions. Geology.

[13] Tajčmanová L, Podladchikov Y, Powell R, Moulas E, Vrijmoed J, Connolly JAD (2014). Grain-scale pressure variations and chemical equilibrium in high-grade metamorphic rocks. J. Metamorph. Geol..

[14] Hobbs BE, Ord A (2017). Pressure and equilibrium in deforming rocks. J. Metamorph. Geol..

[15] Zhong X, Vrijmoed J, Moulas E, Tajčmanová L (2017). A coupled model for intragranular deformation and chemical diffusion. Earth Planet. Sci. Lett..

[16] De Groot SR, Mazur P (1962). Non-Equilibrium Thermodynamics.

[17] Kuiken GDC (1994). Thermodynamics of Irreversible Processes.

[18] Bird RB, Stewart WE, Lightfoot EN (2007). Transport Phenomena.

[19] Venerus DC, Öttinger HC (2018). A Modern Course in Transport Phenomena.

[20] Powell R, Evans KA, Green ERC, White RW (2018). On equilibrium in non-hydrostatic metamorphic systems. J. Metamorph. Geol..

[21] Guo C, Zhang Y (2016). Multicomponent diffusion in silicate melts: SiO2–TiO2–Al2O3–MgO–CaO–Na2O–K2O System. Geochim. Cosmochim. Acta.

[22] Chakraborty, S. & Ganguly, J. Compositional zoning and cation diffusion in aluminosilicate garnets. *Diffusion, atomic ordering and mass transport, Advances in physical geochemistry,* (Ed. Ganguly, J.) vol 8, Springer, Berlin Heidelberg New York Tokyo, pp 120–175 (1991).

[23] Bird RB, Klingenberg DJ (2013). Multicomponent diffusion-A brief review. Adv. Water Resour..

[24] Landau LD, Lifshitz EM (1987). Fluid Mechanics.

[25] Ahrens, T. J. Equation of state. In *High Pressure Shock Compression of Solids.* (Eds. Asay, J.R. & Shahinpoor, M.), 75–113 (Springer, 1993).

[26] Johnson J, Chéret R (1999). Shock waves in solids: An evolutionary perspective. Shock Waves.

[27] Lebon G, Jou D, Casas-Vázquez J (2008). Understanding Non-equilibrium Thermodynamics.

[28] Hobbs B, Ord A (2015). Dramatic effects of stress on metamorphic reactions: COMMENT. Geology.

[29] Truesdell C (1984). Rational Thermodynamics.

[30] Coleman BD, Noll W (1963). The thermodynamics of elastic materials with heat conduction and viscosity. Arch. Ration. Mech. Anal..

[31] Mueller I, Weiss W (2012). Thermodynamics of irreversible processes—past and present. Eur. Phys. J. H.

[32] Gurtin ME, Fried E, Anand L (2010). The Mechanics and Thermodynamics of Continua.

[33] Grmela M, Öttinger HC (1997). Dynamics and thermodynamics of complex fluids. I. Development of a general formalism. Phys. Rev. E.

[34] Öttinger HC, Grmela M (1997). Dynamics and thermodynamics of complex fluids. II. Illustrations of a general formalism. Phys. Rev. E..

[35] Truesdell C (1962). Mechanical basis of diffusion. J. Chem. Phys..

[36] Larché F, Cahn JW (1973). A linear theory of thermochemical equilibrium of solids under stress. Acta Metallurg..

[37] Connolly, J.A.D. The geodynamic equation of state: What and how. *Geochem. Geophys. Geosyst. *10 (2009).

[38] Štípská P, Powell R, White RW, Baldwin JA (2010). Using calculated chemical potential relationships to account for coronas around kyanite: An example from the Bohemian Massif. J. Metamorph. Geol..

[39] Tajčmanová L, Abart R, Neusser G, Rhede D (2011). Growth of decompression plagioclase rims around metastable kyanite from high–pressure felsic granulites (Bohemian Massif). J. Metamorph. Geol..

[40] Tajčmanová L, Vrijmoed J, Moulas E (2015). Grain-scale pressure variations in metamorphic rocks: Implications for the interpretation of petrographic observations. Lithos.

[41] Holland TJB, Powell R (1998). An internally consistent thermodynamic data set for phases of petrological interest. J. Metamorph. Geol..

[42] Fuhrman ML, Lindsley DH (1988). Ternary feldspar modeling and thermometry. Am. Miner..

[43] Merk HJ (1959). The macroscopic equations for simultaneous heat and mass transfer in isotropic, continuous and closed systems. Appl. Sci. Res..

[44] Gibbs JW (1906). The Scientific Papers: Thermodynamics.

