# Supplementary material for: The choice of a thermodynamic formulation dramatically affects modelled chemical zoning in minerals
Source: Sci Rep. 2021 Sep 21;11:18740. doi: 10.1038/s41598-021-97568-x (PMC8455693; doi:10.1038/s41598-021-97568-x)
Supplement: Supplementary file 1 — Supplementary Information 1. [file 41598_2021_97568_MOESM1_ESM.pdf]

The choice of a thermodynamic formulation dramatically affects modelled chemical zoning in minerals

Tajčmanová L.[1*], Podladchikov. Y.[2,3], Moulas E.[4] and Khakimova L.[3,5]

[1] *Institute of Earth Sciences, Heidelberg University, Germany*

[2] *Institute of Earth Science, University of Lausanne, Switzerland*

[3] *Faculty of Mechanics and Mathematics, Moscow State University, Moscow, Russian Federation*

[4] *Institute of Geosciences & Mainz Institute of Multiscale Modeling (M [3]ODEL), Johannes-Gutenberg University of Mainz, Germany*

[5]*Skolkovo Institute of Science and Technology, Moscow, Russian Federation*

Supplementary Material S1

Balance and conservation forms

For species j that diffuse and react within a single phase, the mass balance reads:

$$\frac{\partial \rho \mathrm{x}^j}{\partial t} + \nabla_i\left(\rho \mathrm{x}^j v_i^j\right) = Q^{\mathrm{x}^j} \tag{A1}$$

where ρ is the density of a single phase (in kg·m^{-3}), x^j is the mass fraction of species j in the phase, Q^{x^j} is the volumetric production rate of x (in kg·s^{-1}·m^{-3}) of species j. v_i^j is the velocity (in m/s) of species j with respect to a reference Eulerian frame and repeated subscripts imply summation. Note that balance of mass is formulated with respect to the actual Eulerian state. Summing up over l species yields the equation of total mass conservation.

$$\frac{\partial \rho}{\partial t} + \nabla_i(\rho v_i) = 0 \tag{A2}$$

where we have used that $\sum_{j=1}^{l} Q^{\mathrm{x}^j} = 0$ and the definition of barycentric (mass-averaged) velocity v_i as shown below.

$$v_i = \frac{1}{\rho}\sum_{j=1}^{l} \mathrm{x}^j v_i^j \tag{A3}$$

With the use of v_i, the mass balance for species j (Eq. A1) can be formulated as follows

$$\frac{\partial}{\partial t}\left(\rho \mathrm{x}^j\right) + \nabla_i\left(\rho v_i \mathrm{x}^j\right) + \nabla_i\left(\rho \mathrm{x}^j\left(v_i^j - v_i\right)\right) = Q^{\mathrm{x}^j} \tag{A4}$$

which, after grouping terms, simplifying (using Eq. A2), and substituting the Lagrangian derivative ($\frac{d}{dt} \coloneqq \frac{\partial}{\partial t} + v_i \nabla_i$) becomes:

$$\rho \frac{d\mathrm{x}^j}{dt} + \nabla_i q_i^{\mathrm{x}^j} = Q^{\mathrm{x}^j} \tag{A5}$$

where $q_i^{\mathrm{x}^j} = \rho \mathrm{x}^j\left(v_i^j - v_i\right)$ expresses the mass flux with respect to the barycentric reference frame. Equation A5 is the general mass balance equation in reacting/diffusing systems. In a similar manner, the conservation/balance laws for the total (including all species within a phase) energy (u), momentum (v_j) and entropy (s; all per unit mass) read:

$$\rho \frac{du}{dt} + \nabla_i q_i^u = 0 \tag{A6}$$

$$\nabla_i q_{ij}^{v_j} = 0 \tag{A7}$$

$$\rho \frac{ds}{dt} + \nabla_i q_i^s = Q^s \tag{A8}$$

where Q^s is the production term for entropy and we have considered that kinetic energy contributions are negligible (inertial terms are zero). The fluxes for energy, momentum and entropy are indicated by q_i^u, $q_{ij}^{v_j}$ and q_i^s respectively.

The formulation of Local Thermodynamic Equilibrium (LTE)

Following the notation of classical non-equilibrium thermodynamics (De Groot & Mazur, 1962; Sekerka, 2000; Venerus & Öttinger, 2018; Chen, & Huang, 2021), the LTE hypothesis can be writen as:

$$\rho\frac{du}{dt} = T\rho\frac{ds}{dt} + \frac{p}{\rho}\frac{d\rho}{dt} + \rho\sum_{j=1}^{l}\mu^j\frac{d\mathrm{x}^j}{dt} + \cdots \tag{A9}$$

where μ^j is the chemical potential of species j (in J/kg) and the ellipsis symbol (…) indicates that we have ignored shear work and external forces for simplicity. In the case of a binary system with species α and β, $l = 2$ and $\mathrm{x}^\alpha + \mathrm{x}^\beta = 1$. Note that only $l - 1$ component is independent and therefore $d\mathrm{x}^\beta = -d\mathrm{x}^\alpha$. Thus, A9 can be written as:

$$\rho\frac{du}{dt} = T\rho\frac{ds}{dt} + \frac{p}{\rho}\frac{d\rho}{dt} + \left(\mu^\alpha - \mu^\beta\right)\rho\frac{d\mathrm{x}^\alpha}{dt} + \cdots \tag{A10}$$

For simplicity, deviatoric elastic strain is not considered as a significant contribution to internal energy (Sekerka & Cahn, 2004; Connolly, 2009, errata; Frolov & Mishin, 2010a,b). Under static mechanical equilibrium, the conservation of momentum in the j direction reads:

$$0 = \nabla_i q_{ij}^{v_j} \tag{A11}$$

Multiplying Eq. A11 by v_j and adding it Eq. A10 we obtain (after replacing the respective conservation laws):

$$\begin{aligned} TQ^s = \nabla_i\left(Tq_i^s + v_j q_{ij}^{v_j} + \left(\mu^\alpha - \mu^\beta\right)q_i^\alpha\right) - \nabla_i q_i^u - q_i^s\nabla_i T - q_i^{\mathrm{x}^\alpha}\nabla_i\left(\mu^\alpha - \mu^\beta\right) \\ + \left(\mu^\alpha - \mu^\beta\right)Q^{\mathrm{x}^\alpha} - q_{ij}^{v_j}\nabla_i v_j + p\nabla_i v_i \ldots \end{aligned} \tag{A12}$$

In case of α - albite and β – anorthite, Q^{x^α} is zero. In the classic form of LTE, the fluxes q_i and the source terms are unspecified and they must be chosen so that $Q^s \geq 0$ (see De Groot & Mazur, 1962, p. 22). Thus, by setting $q_i^u = Tq_i^s + v_j q_{ij}^{v_j} + q_i^{\mathrm{x}^\alpha}\left(\mu^\alpha - \mu^\beta\right) + \cdots$ we can write Eq. A12 as:

$$TQ^s = -q_i^s\nabla_i T - q_i^{\mathrm{x}^\alpha}\nabla_i\left(\mu^\alpha - \mu^\beta\right) - q_{ij}^{v_j}\nabla_i v_j + p\nabla_i v_i \ldots \tag{A13}$$

Equation A13 can satisfy entropy production (for $T \geq 0$) if the fluxes are chosen as follows:

$$\begin{gathered} q_i^s = -k\nabla_i T, \qquad q_i^{\mathrm{x}^\alpha} = -K\nabla_i\left(\mu^\alpha - \mu^\beta\right) = -K\nabla_i\left(\frac{\hat{\mu}^\alpha}{M^\alpha} - \frac{\hat{\mu}^\beta}{M^\beta}\right), \\ q_{ij}^{v_j} = p\delta_{ij} - 2\eta(\frac{1}{2}\left(\nabla_i v_j + \nabla_j v_i\right) - \frac{1}{3}\delta_{ij}\nabla_i v_i) \end{gathered} \tag{A14}$$

where k, K and η are positive coefficients, $\hat{\mu}$ is the mol-specific chemical potential (in J/mol) and M is the molecular mass of the endmember (in kg/mol). Note, that in a binary system, there is only one independent diffusional flux. Therefore, there are no cross terms in diffusional fluxes. For simplicity, we do not consider cross terms related to the thermal diffusion because they are negligible for typical geothermal gradients.

It is important to note that a mathematically equivalent expression to the entropy production Q^s using Classical Irreversible Thermodynamics (used here) can also be obtained by the Coleman-Noll procedure (Eq. 70.7 in Gurtin et al. 2010). This means that both, the classical irreversible and the most modern thermodynamics approaches, result in identical expression to ensure that the second law of thermodynamics is not violated.

Additional comment

In Gibbs (1906), chemical equilibrium in fluids is ensured by equality of chemical potentials of the same substance in different phases. Therefore, any molar- or mass-based chemical potential can be used, because both sides of the equality can be normalized by the molecular mass of a given substance (Powell et al., 2018). Here, the present work is an outcome of the appearance of the chemical potential difference in the expression for entropy production (A10) due to the interdependence of mass fraction in a phase. By definition the mass fractions sum up to unity. Thus, the difference cannot be normalized using only one of the molecular masses. Therefore, we cannot simply switch from mass and molar formulations.

Alternatively, without using the chemical potential difference, one can derive similar expressions for diffusion fluxes and entropy production by using the Gibbs-Duhem relation for fluid systems (e.g. Fitts, 1962 for the mass based approach). The use of Gibbs-Duhem relation is required to account for the inter-dependence of chemical potentials in a multicomponent system. Unfortunately, this approach is only correct for fluids that can support no differential stress and pressure gradients in the absence of external forces. For such a system, mechanical equilibrium implies uniform pressure. In fact, the treatment is appropriate for fluids at rest but not for solids at mechanical equilibrium that can have both, pressure jumps between phases as well as pressure gradients within a phase. As a result, Gibbs-Duhem equation would have to have additional terms accounting for pressure gradients and jumps compared to the treatment in Fitts 1962 (page 44, equations 4.24 and 4.26 in Fitts, 1962). In other words, the expressions of Fitts are correct (for fluids at rest) but not appropriate for the particular case, we aim to solve in our work. For the full approach, the derivation based on chemical potential difference provided earlier in this Supplementary material S1 is required.

More specifically, individual chemical potentials would have additional terms to account for the pressure gradient in the generalized Gibbs-Duhem relation. Therefore, the chemical potential for solids under pressure gradients would have to be different compared to the classical chemical potential expression for fluids. On the contrary, the chemical potential difference has this additional term canceled. Therefore, the difference in chemical potential approach is used here to exclude the need to discuss the generalized Gibbs-Duhem relation under pressure gradients. The present approach is similar to other modern thermodynamic treatments of multicomponent diffusion for fluids and solids (e.g. Taylor & Krishna, 1993; Kuiken, 1994; Sekerka, 2000; Gurtin et al., 2010;Venerus & Öttinger, 2018)

Finally, when pressure gradients are not present, Gibbs-Duhem relation can be used for the dependent chemical potential, and similar expressions for entropy production can be derived without the use of chemical potential difference sensitive to mass and moles conversion. Therefore, in such a system, mass- and molar-based formulations are equivalent to treat the multicomponent diffusion problems. Mathematically, zero pressure gradients make molar- (Eq. 8 in the main text) and mass-based (Eq. 10 in the main text) formulations identical. Under zero pressure gradients, both formulations would predict no chemical zoning at equilibrium.

References:

Chen, H. & Huang, HY. Modeling and simulation of the non-equilibrium process for a continuous solid solution system in lithium-ion batteries. International Journal of Solids and Structures 212, 124-142 (2021).

Connolly, JAD. The geodynamic equation of state: what and how. Geochemistry, Geophysics, Geosystems 10 (2009).

De Groot, S.R., & Mazur, P. Non-equilibrium thermodynamics. New York, Dover, 510 pp (1962).

Fitts, D. D. Nonequilibrium Thermodynamics: A phenomenological theory of irreversible processes in fluid systems. McGraw-Hill, 192 pp (1962).

Frolov, T., & Mishin, Y. Effect of non-hydrostatic stresses on solid fluid equilibrium. I. Bulk thermodynamics. Physical Review B, 82, 174113 (2010a).

Frolov, T., & Mishin, Y. Effect of non-hydrostatic stresses on solid fluid equilibrium. II. Interface thermodynamics. Physical Review B, 82, 174114 (2010b).

Gibbs, J.W. The Scientific Papers: Thermodynamics. Longmans, Green and co, London, 434 pp (1906).

Gurtin., M. E., Fried, E. & Anand, L. The Mechanics and Thermodynamics of Continua. Cambridge (2010).

Kuiken, G. D. C. Thermodynamics of Irreversible Processes. Wiley, New York, 458 pp (1994).

Powell, R., Evans, K.A., Green, E.C.R. & White, R.W. On equilibrium in non-hydrostatic metamorphic systems. Journal of Metamorphic Geology, 36, 419-438 (2018).

Sekerka R.F. Notes on diffusion with volume change. Carnegie Mellon University Pittsburgh, Pennsylvania 15213-3890 pp 21 (2000). https://userpages.umbc.edu/~dfrey1/ench630/diffusionvolumechange.pdf

Sekerka, R. F., & Cahn, J. W. Solid–liquid equilibrium for non-hydrostatic stress. Acta Materialia, 52(6), 1663–1668 (2004).

Taylor, R. & Krishna, R. Multicomponent mass transfer. Wiley, New York, 616 pp (1993).

Venerus, D. C. & Öttinger, H. C. A modern course in transport phenomena. Cambridge University Press, 508 pp (2018).
