# Supplementary material for: The choice of a thermodynamic formulation dramatically affects modelled chemical zoning in minerals
Source: Sci Rep. 2021 Sep 21;11:18740. doi: 10.1038/s41598-021-97568-x (PMC8455693; doi:10.1038/s41598-021-97568-x)
Supplement: Supplementary file 2 — Supplementary Information 2. [file 41598_2021_97568_MOESM2_ESM.pdf]

The choice of a thermodynamic formulation dramatically affects modelled chemical zoning in minerals

Tajčmanová L.[1*], Podladchikov. Y.[2,3], Moulas E.[4] and Khakimova L.[3,5]

[1] *Institute of Earth Sciences, Heidelberg University, Germany*

[2] *Institute of Earth Science, University of Lausanne, Switzerland*

[3] *Faculty of Mechanics and Mathematics, Moscow State University, Moscow, Russian Federation*

[4] *Institute of Geosciences & Mainz Institute of Multiscale Modeling (M [3]ODEL), Johannes-Gutenberg University of Mainz, Germany*

[5] *Skolkovo Institute of Science and Technology, Moscow, Russian Federation*

Supplementary Material S2

Sensitivity of model results to non-ideality of thermodynamic solution models and the comparison with the Larché & Cahn theory

The results presented in the main text use the ideal molecular mixing for simplicity reasons. In this supplement, we will show the comparison of this model to more sophisticated formulations that include i) ideal but with mixing on sites (Fig. S2.1), ii) non-ideal with mixing on sites after Fuhrman & Lindsley (1988; Fig. S2.2) and iii) the generalization of chemical potentials in the framework of thermodynamics of stressed solids as it was pioneered by Larché & Cahn (1973; Fig. S2.3). For notation reasons, we will refer to the respective models as $ideal_1$ for the simple ideal molecular mixing model, $ideal_2$ for the ideal mixing on sites (e.g. Cemič, 2005, p. 209-213) and *LC* for the Larché & Cahn (1973) generalization.

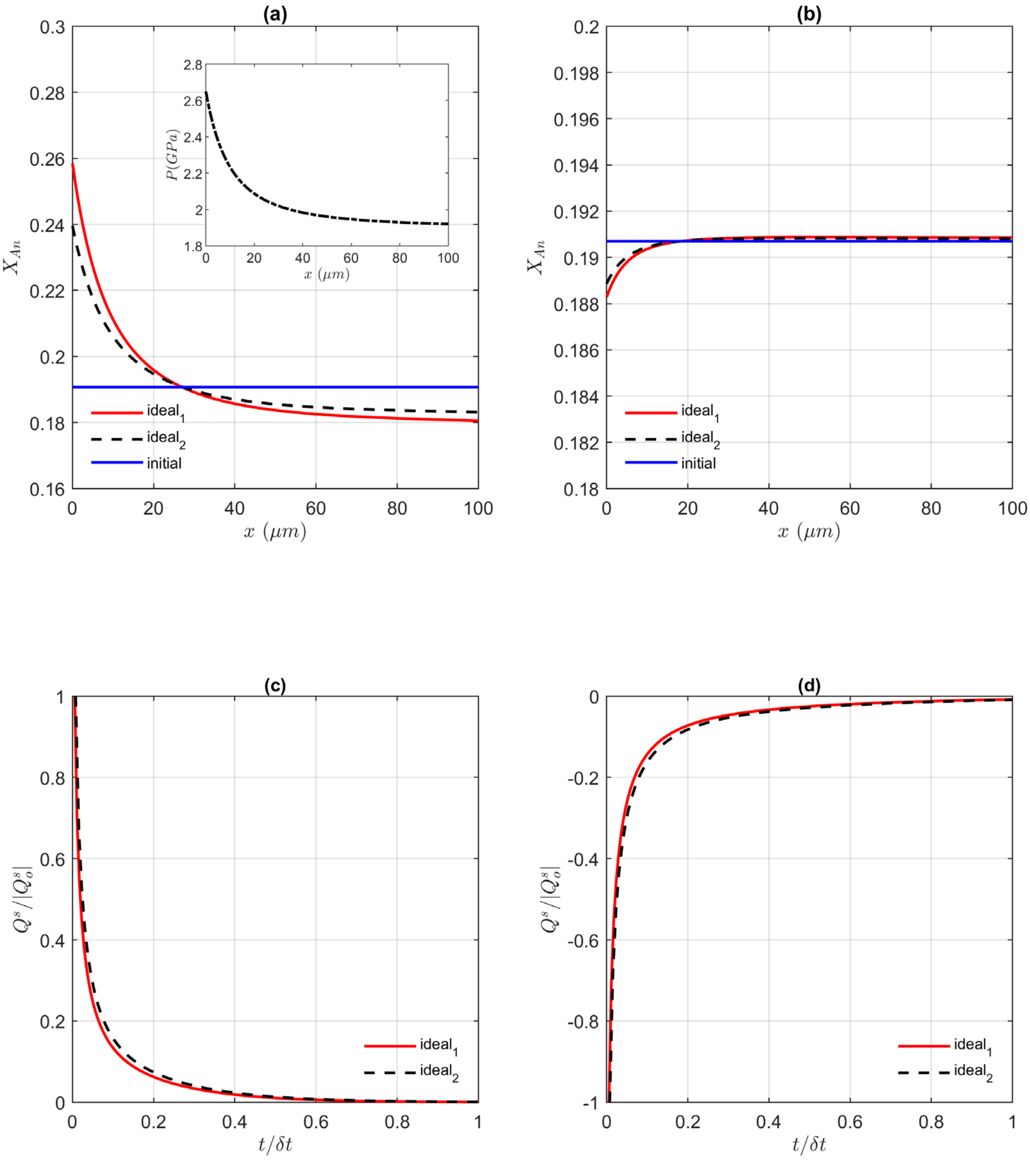

Figure S2.1. Comparison between two different ideal solid solution formulations ($ideal_1$ and $ideal_2$). The rest of the figure is as in Fig. 4 of the main text.

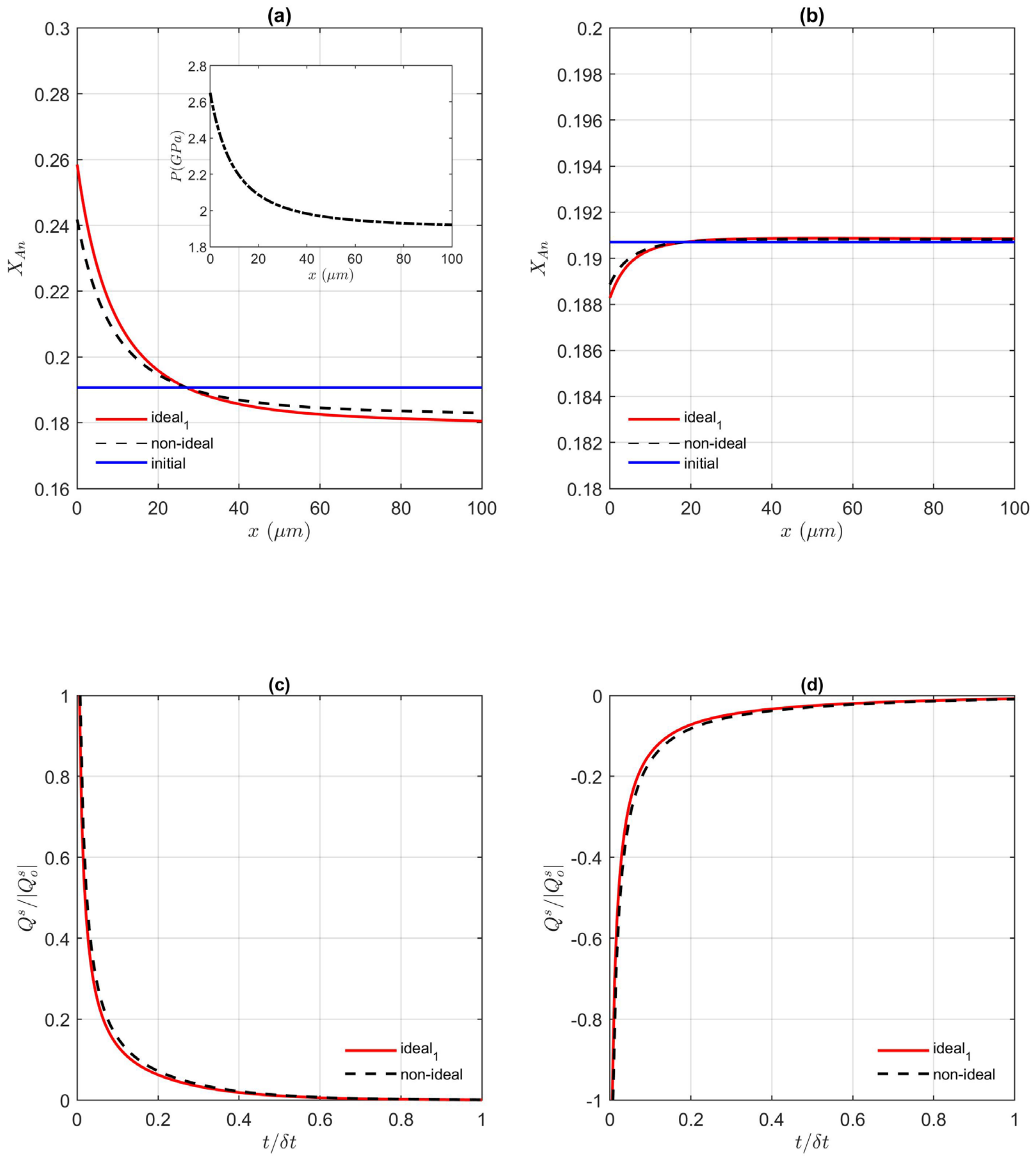

Figure S2.2. Comparison between the ideal (ideal$_1$) and the non-ideal solid solution formulations. This figure is identical to Fig. 4 of the main text.

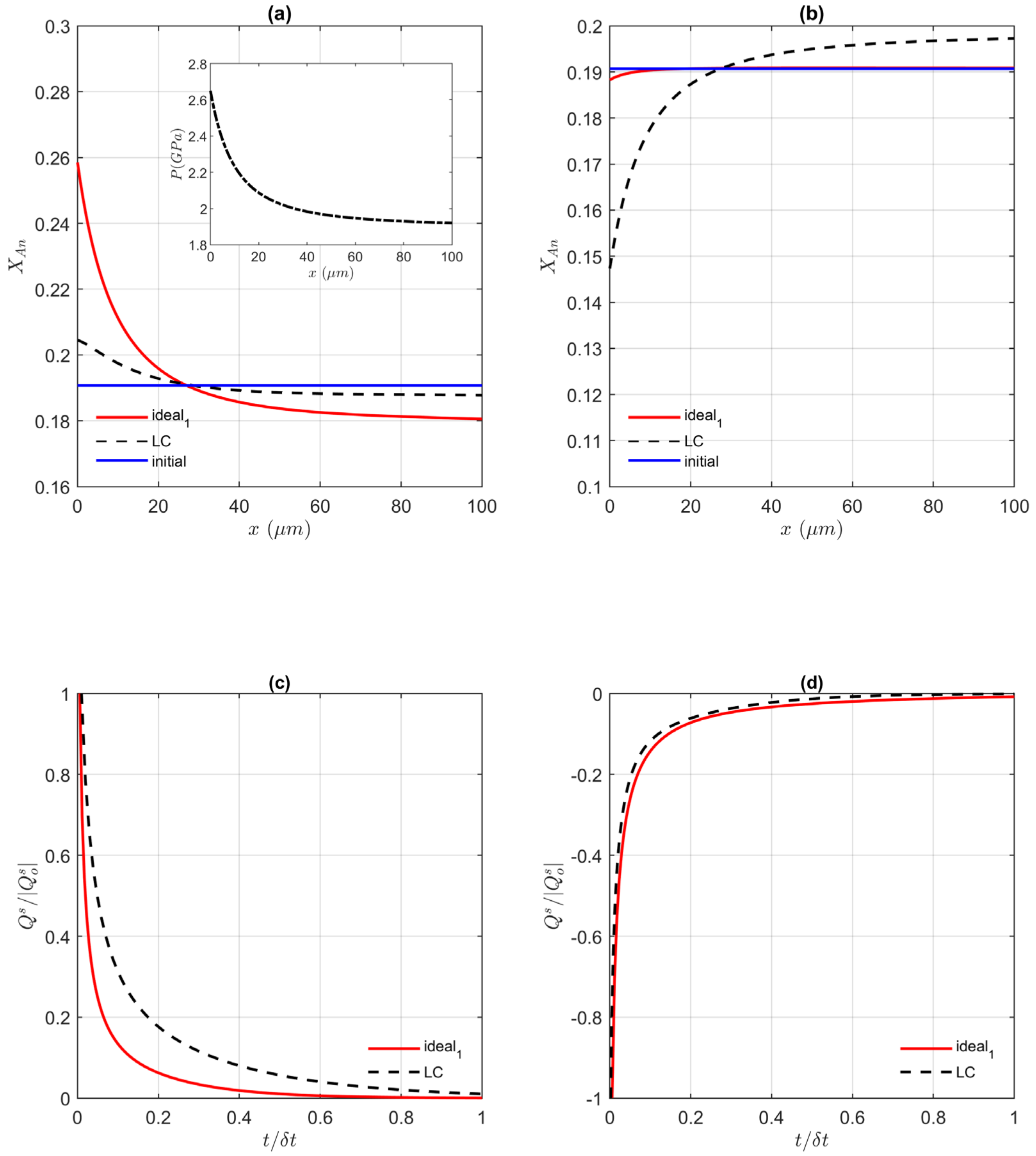

Figure S2.3. Comparison between the ideal (ideal$_1$) and the generalized chemical potential following Larché & Cahn theory (LC). The rest of the figure is as in Fig. 4 of the main text.

References:

Fuhrman, M.L. & Lindsley, D.H., Ternary feldspar modeling and thermometry. *American Mineralogist* 73, 201–215 (1988).

Cemič, L. Thermodynamics in Mineral Systems, Springer (2005)

Larché F. & Cahn. J. W. A linear theory of thermochemical equilibrium of solids under stress. Acta Metall 21, 1051–1063 (1973).
